# Anesthesia for deep brain stimulation system implantation: adapted protocol for awake and asleep surgery using microelectrode recordings

**DOI:** 10.1007/s00701-021-05108-3

**Published:** 2022-02-25

**Authors:** Jan Vesper, Bernd Mainzer, Farhad Senemmar, Alfons Schnitzler, Stefan Jun Groiss, Philipp J. Slotty

**Affiliations:** 1 Department of Functional Neurosurgery and Stereotaxy, Medical Faculty, Heinrich-Heine University Düsseldorf, Moorenstr. 5, 40225 Düsseldorf, Germany; 2 Department of Anesthesia and Intensive Care Medicine, Medical Faculty, Heinrich-Heine University Düsseldorf, Düsseldorf, Germany; 3 Department of Neurology & Institute for Clinical Neuroscience and Medical Psychology, Medical Faculty, Heinrich-Heine University Düsseldorf, Düsseldorf, Germany

**Keywords:** Deep brain stimulation, Parkinson’s disease, Asleep implantation, Awake implantation, Anesthesia

## Abstract

**Purpose:**

Deep brain stimulation (DBS), an effective treatment for movement disorders, usually involves lead implantation while the patient is awake and sedated. Recently, there has been interest in performing the procedure under general anesthesia (asleep). This report of a consecutive cohort of DBS patients describes anesthesia protocols for both awake and asleep procedures.

**Methods:**

Consecutive patients with Parkinson’s disease received subthalamic nucleus (STN) implants either moderately sedated or while intubated, using propofol and remifentanil. Microelectrode recordings were performed with up to five trajectories after discontinuing sedation in the awake group, or reducing sedation in the asleep group. Clinical outcome was compared between groups with the UPDRS III.

**Results:**

The awake group (*n* = 17) received 3.5 mg/kg/h propofol and 11.6 μg/kg/h remifentanil. During recording, all anesthesia was stopped. The asleep group (*n* = 63) initially received 6.9 mg/kg/h propofol and 31.3 μg/kg/h remifentanil. During recording, this was reduced to 3.1 mg/kg/h propofol and 10.8 μg/kg/h remifentanil. Without parkinsonian medications or stimulation, 3-month UPDRS III ratings (ns = 16 and 52) were 40.8 in the awake group and 41.4 in the asleep group. Without medications but with stimulation turned on, ratings improved to 26.5 in the awake group and 26.3 in the asleep group. With both medications and stimulation, ratings improved further to 17.6 in the awake group and 15.3 in the asleep group. All within-group improvements from the off/off condition were statistically significant (all ps < 0.01). The degree of improvement with stimulation, with or without medications, was not significantly different in the awake vs. asleep groups (ps > 0.05).

**Conclusion:**

The above anesthesia protocols make possible an asleep implant procedure that can incorporate sufficient microelectrode recording. Together, this may increase patient comfort and improve clinical outcomes.

## Introduction

Deep brain stimulation (DBS) is an effective treatment for neurodegenerative disorders with advanced motor symptoms, such as Parkinson’s disease (PD), essential tremor (ET), and dystonia. Treatment involves placing electrodes into basal ganglia structures, targeting the subthalamic nucleus (STN), internal segment of the globus pallidus (GPi), or the ventral intermediate nucleus of the thalamus (VIM) [2, 3]. Treatment may be unilateral or bilateral. Leads are then connected to a subcutaneously implanted pulse generator located in the chest or abdominal region. After programming, stimulation achieves functional inhibition of the overactive neural pathways, improving symptoms of on/off motor fluctuation, tremor, rigidity, bradykinesia, and/or dyskinesia [36].

Leads are placed in a stereotactic procedure using atlas coordinates supplanted with detailed preoperative imaging and/or intraoperative microelectrode recordings (MER) of the brain [12, 35]. According to procedures commonly employed for the past quarter century, DBS patients are awake, although sedated and treated with local anesthetics, during the procedure. This enables MER for the purpose of identifying the optimal physical target and making fine adjustments in lead location. The patient’s cooperation is required during this testing, in order to provide verbal or other feedback regarding cessation of symptoms and/or the appearance of side effects [26]. Quantitative aspects of MER can, however, be assessed without the patient’s cooperation, such as assessment of bursting patterns to identify STN neurons and firing rates to distinguish GPi neurons from those of the external globus pallidus (GPe) [17, 20].

Awake DBS procedures can be stressful and are associated with high burden for PD patients since surgery needs to be performed in the medical off state after cessation of parkinsonian medications. Furthermore, patients may not be fully cooperative or accurate in their feedback due to interference of the sedatives [21, 38]. Awake procedures may extend the duration of surgery and increase the costs, although findings vary on this matter [13, 23]. Taking another approach, DBS implantations can also be completed in asleep patients, that is, under full general anesthesia. Clinical outcomes of awake and asleep lead placements have generally been equivalent [7, 8, 16, 31, 34, 37], albeit with small sample sizes and high patient heterogeneity in some comparative trials. The equivalence of asleep procedures, together with the face validity of increased patient comfort, has led to a shifting of perspectives that makes asleep DBS procedures the preferred approach [6].

When lead placement is solely reliant on imaging during asleep procedures, it can be argued that this limits the precision of targeting. MER may not be incorporated into asleep DBS procedures due to the potential for anesthetics to suppress or alter neuronal activity [5, 17, 20], and the surgeon may prefer to have the conscious cooperation of the patient. Asleep-awake-asleep procedures have been developed to address this, in which deeper sedation is initially induced but discontinued during MER to allow the patient to wake up to the point of cooperation, and then resumed for the completion of the procedure [18, 33]. This approach may, however, see some patients who are slow to rouse from sedation or remain too groggy to participate in the MER feedback. Another option, described in a previous report from our group [4, 31], is to induce full intubated general anesthesia and to temporarily reduce the anesthesia during the MER process to allow the observation of some indicators such as the appearance of discrete muscle twitching due to pyramidal tract stimulation.

A previous report from our group has shown that asleep DBS procedures are feasible and result in clinical outcomes that are as good or better than those of awake procedures [4]. A caveat of asleep DBS procedures is that anesthesia dosage must be carefully titrated to allow sufficient brain activity for microelectrode recordings. This report describes the anesthesia protocol for both awake and asleep DBS procedures at a high-volume center to achieve both options safely and effectively. The patients in this study are a single center sub-group from a recent report [30].

## Methods

### Study design

Patients were enrolled as part of a larger prospective multi-center study [30]. This report includes all consecutively enrolled patients from a single center (Center of Neuromodulation in Düsseldorf, Germany). Separate from the larger study, this report concerns data captured to establish procedural recommendations for anesthesia dosage and timing in DBS microelectrode recording (MER). Patients were enrolled between April 2016 and May 2019. The study had the oversight of the institutional ethics committee and was publicly registered (study number 5379R, registration-ID 2,019,095,251). Participants gave their written informed consent before the initiation of study procedures.

### Patients

Patients were adults between the ages of 32 and 83, capable of understanding and consenting to study procedures, and suitable candidates for de novo bilateral DBS implantations targeting the subthalamic nucleus (STN-DBS) for Parkinson’s disease with the Infinity system (Abbott). Patients were not considered for DBS procedures on the basis of high-risk comorbidities or DBS contraindications that would present a risk to safety.

### Surgical procedures

There were two sets of patients: those chosen for asleep surgery and those chosen for awake surgery. This determination was made clinically by the multidisciplinary team including neurologists and neurosurgeons in consultation with the patient and family members, at a pre-surgery planning conference. Decisions took into account the patient’s general condition, age, and patient’s preference. All procedures were conducted in medication off state, with all dopaminergic medication discontinued at least 12 h before surgery, except for subcutaneous apomorphine, which was discontinued in the morning of surgery [32]. Generally, the surgical procedures are consistent with standard practices and have been described previously [1, 4]. Stereotactic planning was performed using preoperative MRI including T1/T2/FLAIR sequences and contrast enhanced stereotactic CT. The head was fixed on a stereotactic Leksell frame. Infusions of propofol and remifentanil were used in all patients, as described in the next section, using dosing that achieved moderate sedation in the awake group and complete general anesthesia (requiring endotracheal intubation) in the asleep group. Stereotactic targeting of the STN was performed the day before surgery using MRI sequences, according to AC PC line, anatomical structures, and Schaltenbrand Wahren atlas coordinates. Directional DBS leads (0.5 mm [small space for STN], Infinity DBS system; Abbott) were used.

All but two patients received MER with up to five microelectrodes. The two patients had just macroelectrode implantation due to revision surgery. We included them because of the intraoperative testing under the anesthesia protocol as described in the “Methods” section. Intraoperatively microstimulation via MER electrodes (Inomed GmbH, Emmendingen, Germany) was used [14, 28]. First, testing at low frequency stimulation at 4 Hz with 210–500 μs pulse width and up to 6 mA intensity followed by effective stimulation using 60 μs pulse width, 130 Hz frequency and stimulation amplitude up to 5 mA. In the awake group, recordings were completed after a temporary discontinuation of sedation (10–15 min). Because patients were fully awake and could follow instructions, clinical effects such as resolution of tremor/rigidity symptoms and the appearance of adverse events such as dysarthria, muscle contraction, eye movement, and dysesthesia were evaluated. For patients in the asleep group, sedation was reduced for the sake of optimal MER discharges in the recorded brain activity but without regaining any awareness or requiring extubation. Low frequency stimulation using the macrocontact of the micro-/macroelectrode was first performed to evaluate the threshold for irregular muscular activity as an indicator for stimulating the internal capsule. Then, 130 Hz stimulation was tested to show potential side effects.

Due to the level of anesthesia, contralateral muscle contractions and other side effects (e.g., from activation of the internal capsule) were the only observations. In both groups, the electrophysiological recordings provided information about correct electrode positioning as was also assessed by intraoperative x-ray and confirmed by intraoperative stereotactic CT at the end of surgery. The pulse generator (Infinity DBS system; Abbott) was subsequently implanted under general anesthesia within 3 days or immediately after electrode insertion.

### Assessments and analysis

Since all included patients underwent surgery, there was no loss of follow-up. Anesthesia administration was captured for all patients. Motor function after 3 months of treatment was assessed for PD by the motor domain of the unified PD rating scale (UPDRS III) under three conditions: without parkinsonian medications or stimulation (meds off/stim off), without medications but with stimulation turned on (meds off/stim on), and with medications and stimulation turned on (meds on/stim on). Descriptive statistics (means, standard deviations [± SD]) were calculated. The degree of UPDRS III improvement from meds off/stim off was compared to both meds off/stim on and meds on/stim on, forming the primary outcome criteria. Differences of UPDRS III within each of the groups were analyzed with Friedman tests and post hoc Dunn tests. Statistical power for the samples was calculated post hoc. GraphPad Prism™ was used for statistical analysis. The significance level was set at *p* < 0.05.

## Results

### Patients

The study enrolled 80 patients, all with PD. Of these, 17 were implanted with awake procedures. This group included 15 men and 2 women with an average age of 59.1 years (range: 38–78). The remaining 63 patients were implanted while asleep. The group included 39 men and 24 women with an average age of 64.1 years (range 40–79 years). Baseline characteristics of the patients were similar between groups (Table 1).

**Table 1:** Baseline characteristics

	Awake (*n* = 17)	Asleep (*n* = 63)
Age, years (mean)	59.1	64.1
Gender (% male)	88%	62%
Duration of PD (mean)	8.7	9.9
Preoperative Hoehn & Yahr stage (mean, range)	2.2 (2–2.5)	2.4 (1–4)
Preoperative levodopa equivalent daily dose (mean, range)	575 (200–1550)	644 (200–2000)
Preoperative UPDRS III score, off (mean, range)	41.8 (29–60)	39.7 (15–76)

### Anesthesia dosage

#### Awake group

Propofol was delivered at 3.5 (± 2.1) mg/kg/h (range: 0.8–6.7). Remifentanil was administered at a rate of 11.6 (± 6.5) μg/kg/h (5.0–25.0). During the test phase, the administration of anesthesia was stopped for both propofol and remifentanil. The average surgery duration (OR time from skin incision to last stitches) was 153 min (105–181), and subjects were under anesthesia for all of that time, but for the short cessation of medications during testing. No other anesthetics were used during surgery. A mean of 3.3 (± 0.99) MER trajectories was used per side, in all but two patients. In the large majority of cases, the central (planned) MER trajectory was used (85.3% of the awake patients and 70.6% of the asleep patients; see Table 2).

**Table 2:** Microelectrode recording trajectories on each side

Trajectory	Awake			Asleep		
	Left (*n* = 17)	Right (*n* = 17)	Total (*n* = 34)	Left (*n* = 63)	Right (*n* = 63)	Total (*n* = 126)
Central (planned) *n (%)*	16 (94.1%)	13 (76.5%)	29 (85.3%)	45 (71.4%)	44 (69.8%)	89 (70.6%)
Anterior *n (%)*	0 (0%)	1 (5.9%)	1 (2.9%)	6 (9.5%)	6 (9.5%)	12 (9.5%)
Posterior *n (%)*	0 (0%)	0 (0%)	0 (0%)	3 (4.8%)	0 (0%)	3 (2.4%)
Medial *n (%)*	0 (0%)	1 (5.9%)	1 (2.9%)	1 (1.6%)	3 (4.8%)	4 (3.2%)
Lateral *n (%)*	1 (5.9%)	1 (5.9%)	2 (5.9%)	5 (7.9%)	5 (7.9%)	10 (7.9%)
Other *n (%)*	0 (0%)	1 (5.9%)	1 (2.9%)	3 (4.8%)	5 (7.9%)	8 (6.3%)

#### Asleep group

Initial dosage during the fully asleep phase used propofol at 6.9 (± 1.5) mg/kg/h (2.7–10.7) and remifentanil at 31.3 (± 8.0) μg/kg/h (10.0–50.0), respectively. During the test phase, this was reduced, allowing for microelectrode recording, to 3.1 (± 1.0) mg/kg/h (0.1–6.7) propofol and 10.8 (± 2.5) μg/kg/h (4.0–20.0) remifentanil (Table 3). The average surgery duration was 142 min (53–205), and subjects were under anesthesia for all of that time. No other anesthetics were used during surgery. MER recording was used for determination of the borders of the STN and yielded useful information for all patients across a mean of 3.9 trajectories (± 0.77) per side. In addition, clinical testing for side effects provided a reliable marker for the proximity to the internal capsule.

**Table 3:** Anesthesia protocols during awake and asleep procedures

		Awake (*n* = 17)		Asleep (*n* = 63)	
		Implant phase	Test phase	Asleep phase	Test phase
Propofol (mg/kg/h)	Mean (SD) Range	3.5 (2.1) 0.8–6.7	None	6.9 (1.5) 2.7–10.7	3.1 (1.0) 0.1–6.7
Remifentanil (μg/kg/h)	Mean (SD) Range	11.6 (6.5) 5.0–25.0	None	31.3 (8.0) 10.0–50.0	10.8 (2.5) 4.0–20.0

### Clinical outcomes for PD patients (UPDRS III ratings)

**Fig. 1:**
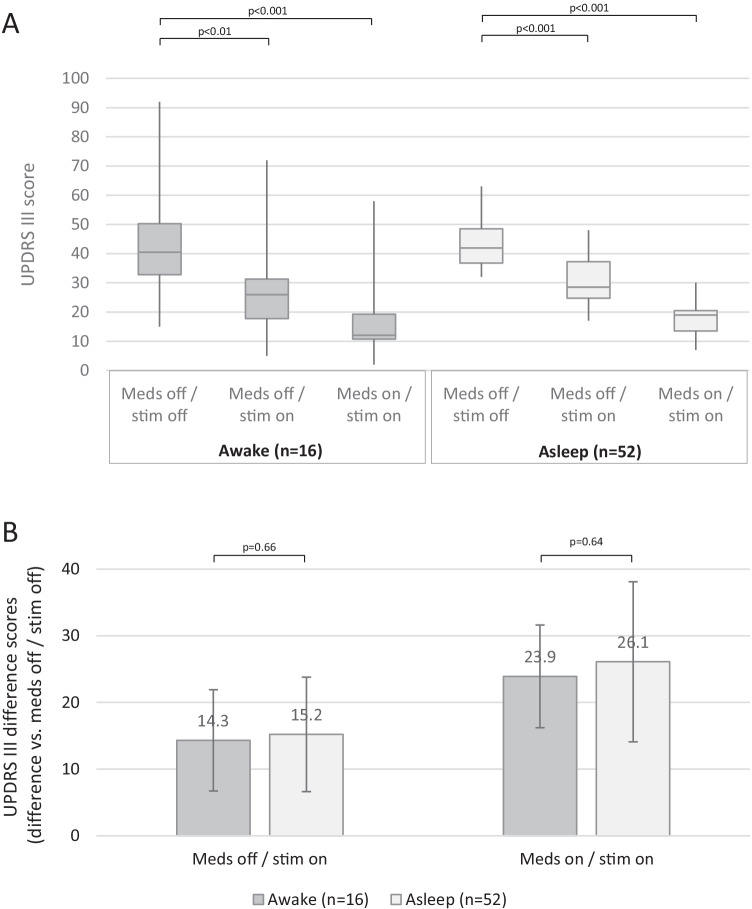
**A** Box plots showing UPDRS III scores at 3 months in the awake and asleep groups under three conditions: without medications or stimulation (meds off/stim off), without medications but with stimulation turned on (meds off/stim on), and with medications and stimulation turned on (meds on/stim on). Comparisons and results of hypothesis testing are indicated. **B** UPDRS III difference scores, relative to meds off/stim off, for the meds off/stim on and meds on/stim on conditions for both the awake and asleep groups. Bar heights and error bars represent means and standard deviations. Comparisons and results of hypothesis testing are indicated

UPDRS III ratings were analyzed in 16 awake and 52 asleep patients (a small number of patients were unable to complete testing). After 3 months of treatment, outcomes were favorable in both groups. Without medications or stimulation (meds off /stim off), ratings were 40.8 (± 9.7) in the awake group and 41.4 (± 15.1) in the asleep group. Without medications but with stimulation turned on (meds off/stim on), ratings improved to 26.5 (± 8.7) in the awake group and 26.3 (± 12.4) in the asleep group. With both medications and stimulation (meds on/stim on), ratings improved further to 17.6 (± 6.6) in the awake group and 15.3 (± 9.5) in the asleep group. Friedman tests revealed significant differences between the conditions for both groups (awake: *X*^2^ = 28.5, *p* < 0.0001, asleep: *X*^2^ = 103.0, *p* < 0.0001), and statistical power was higher than 90% within each group. Post hoc Dunn tests showed statistically significant improvements relative to the meds off/stim off condition for both meds off/stim on (awake: *p* < 0.01, asleep: *p* < 0.001) and meds on/stim on (asleep: *p* < 0.001, asleep: *p* < 0.001) (Fig. 1A). The degree of improvement (difference scores, relative to meds off/stim off) was not different between the awake vs. asleep groups for either meds off/stim on (awake 14.3 [± 7.6], asleep 15.2 [± 7.7], *p* = 0.66) or meds on/stim on (awake 23.9 [± 8.6], asleep 26.1 [± 12.0], *p* = 0.64) (Fig. 1B).

Stimulator implantation usually took place 2 days after electrode insertion. The electrodes were externalized for scientific reasons to undergo MEG examinations. All but two patients agreed to participate; the two were implanted in an all-in-one procedure. The intended level of anesthesia was maintained throughout the procedures for all patients. A single patient in the awake group raised his hand during the test phase with shallow sedation (as previously agreed in case of any perception) but did not show any cardio-vascular stimulation nor any recollection later. No serious complications occurred, i.e., hemorrhages or mechanical complications. In two cases, both in the asleep group, infections developed by postoperative month two. Both required stimulator revisions, followed by 6 weeks of specific antibiotic treatment and replacement of the stimulator 4 weeks later. Dopaminergic medication was adapted within the following 6–12 weeks. After 12 weeks, patients returned for a final adaptation of the stimulation setting.

## Discussion

Some reports of asleep procedures for DBS implantation describe targeting based solely on imaging [6, 9, 24]. Generally, MER is not attempted in asleep procedures due to the depth of anesthesia and its effects on neuronal recordings [34]. However, some argue that because it requires multiple brain penetrations, MER increases the risk for intracranial hemorrhage and other complications [7, 25]. The converse position is that careful MER during asleep implantations make for better quality of targeting [26, 27, 29]. As previously shown by our team, by adapting the number of trajectories by respecting the individual anatomical condition of the patient, we did not result in any hemorrhage in our patient cohort [14]. Other work completed by our group has shown the value of using MER during asleep procedures to identify and avoid the pyramidal tract [28].

Controlled anesthesia for intraoperative recordings during STN DBS implantation surgery was first described in 2014 [11]. However, this procedure requires replacement of propofol with sevoflurane, a halogenated gas. Here, we have described minor modifications to the standard DBS implant anesthesia protocol by reducing the propofol and remifentanil dosages considerably, to allow observations of motor activation without bringing the patient out of anesthesia. Those medications were used because they are appropriate for anesthesia induction and maintenance in neurosurgery. In patients with PD, propofol is recommended on balance as an induction agent, although the potential development of dyskinesias should be monitored [10]. No anesthesia-related clinical issues were noted, and clinical outcomes in the asleep group were equivalent or better than those of the awake group. Recently, we could also show that our modified anesthesia protocol may increase therapeutic window compared to conventional awake surgery [31]. Together, this supports the safety and effectiveness of this approach. The use of MER is important to maintain a reasonable outcome in STN DBS; however, the type of anesthesia does not correlate with the outcome [19]. Even more, by using asleep surgery, operating room time can be shortened (including by creating the option for a single procedure within a similar OR setting for both groups), and related complications, like frontal air assumption, can be avoided [15].

Only a minority of PD patients who are eligible for DBS is deciding for themselves to undergo surgery. One of the major reasons to refuse surgery is the fear of having awake brain surgery [22]. Whether asleep DBS reduces the general resistance to undergo DBS within the patient community is a matter of further evaluation.

Limitations of this study include the assessment of UPDRDS-III scores in a subset of subjects (16 of 17 awake and 52 of 63 asleep patients, due to inability to complete testing secondary to fatigue or refusal to temporarily discontinue parkinsonian medications). Although this smaller sample size, as well as the difference in samples sizes between the groups, does limit the generalizability of the data, hypothesis testing was sufficiently robust since statistical power was above 90% for both groups. Another limitation stems from the non-randomized nature of the study design; the choice of sedation versus general anesthesia can introduce selection bias. This was not systematically controlled for but presumably would reflect the larger clinical population in which this choice is offered. Another possible limitation is that selection bias of subjects cannot be ruled out, although therapy-related bias is mitigated by all surgeries having been performed by one surgeon using the same stimulation systems. Achieving long-term data was not the intention of this study nor the PROGRESS trial; even so, the larger study’s timelines proceeded through 6 months, and similar trends in UPDRS scores were observed [30].

In summary, this report describes the anesthesia protocol for minor modifications to the DBS implant workflow that makes possible an asleep implant procedure without sacrificing the option of MER. DBS implantations in an asleep procedure is as good as under awake conditions. This requires only a single procedure and may increase patient comfort. A subsequent report will describe the electrophysiological recordings from both the awake and asleep groups.

## Data Availability

Not applicable.
